# Cognitive impairment in bipolar disorder in comparison to mild cognitive impairment and dementia: a systematic review

**DOI:** 10.47626/2237-6089-2021-0300

**Published:** 2022-11-09

**Authors:** Mario Simjanoski, Aidan McIntyre, Flavio Kapczinski, Taiane de Azevedo Cardoso

**Affiliations:** 1 Mood Disorders Program Department of Psychiatry and Behavioural Neurosciences McMaster University Hamilton ON Canada Mood Disorders Program, Department of Psychiatry and Behavioural Neurosciences, McMaster University, Hamilton, ON, Canada.; 2 Neuroscience Graduate Program McMaster University Hamilton ON Canada Neuroscience Graduate Program, McMaster University, Hamilton, ON, Canada.

**Keywords:** Bipolar disorder, mild cognitive impairment, dementia, cognitive impairment, systematic review

## Abstract

**Objective:**

To conduct a systematic review to describe cognitive abilities in bipolar disorder (BD) in comparison to cognitive abilities in mild cognitive impairment (MCI) and dementia.

**Methods:**

A literature search was performed with no year or language restrictions. The search yielded 1,461 articles, with 1,261 remaining after removal of duplicates, five of which were suitable for the systematic review: two for the comparison between BD and MCI and three comparing BD and dementia.

**Results:**

Analyses from our systematic review showed that euthymic individuals with BD present impairments in cognitive domains such as attention and executive functioning, motor skills, conceptual thinking, and visuo-spatial abilities that are equally severe as or more severe than the impairments observed in individuals with MCI. In contrast, studies comparing BD and dementia indicated that Alzheimer’s disease (AD) dementia and behavioral variant frontotemporal dementia (bvFTD) both showed greater cognitive deficits than BD during euthymia, whereas BD during a mood episode demonstrated higher cognitive impairments than bvFTD.

**Conclusion:**

Findings from our systematic review suggest that cognitive impairments in euthymic BD fall into a range between the impairments seen in MCI and those seen in dementia. More studies are needed to analyze these comparisons, while also focusing on comparing different clinical stages of BD with MCI and dementia to analyze the progression of the clinical course and cognitive dysfunction in BD.**PROSPERO registration ID:** CRD42020150412

## Introduction

Bipolar disorder (BD) is a chronic and recurrent disorder characterized by episodes of mood oscillation.^1^ The lifetime prevalence ranges from 0.4 to 2.4% worldwide, varying according to the subtype of the disorder.^2^ BD is known to affect individuals’ overall functioning and quality of life.^3-6^ Additionally, a subset of patients with BD experience neurocognitive impairment.^3,6^ Many features, such as the number and length of mood episodes, childhood trauma, and severity of manic and depressive symptoms can influence the cognitive abilities of individuals with BD.^6^ These deficits are present in several cognitive domains, such as attention, verbal learning, mental flexibility, and memory.^7,8^

Cognitive dysfunction has become a recognized feature of BD, evident across all mood states of the disorder.^9,10^ Although it is hard to estimate the prevalence of cognitive impairments in individuals with BD, a study by Douglas et al.^11^ suggested that 64.4% of patients with BD during a depressive episode, and 57.1% of euthymic individuals with BD demonstrate impairments across one or more cognitive domains. Differences in cognitive abilities between BD and healthy individuals are noticeable and widely reported,^12-14^ but the extent to which cognitive impairments in BD resemble those in other cognitive disorders remains unclear.

Cognitive dysfunction associated with BD increases with the rise in number and length of mood episodes, where individuals demonstrate more severe cognitive impairments in later stages of the disorder.^15,16^ Cognitive impairment in late-stage BD raises questions about links to other cognitive disorders. The association between BD and dementia due to the development of cognitive symptoms in BD has raised significant concerns.^17^ One of the largest studies of BD and dementia found that the rate of admission for dementia among individuals with BD increased by 6% with each additional mood episode that they experienced in their lifetime.^18^ There are also reports implying that a previous diagnosis of BD increases the risk of a dementia diagnosis by 2.36 times,^17,19^ and a more recent meta-analysis reported individuals with BD to be 2.96 times more likely to develop dementia in comparison to controls.^20^ These findings suggest that a diagnosis of BD is a serious risk factor for dementia.

Furthermore, an intriguing comparison that has not been extensively studied is cognitive performance in BD relative to mild cognitive impairment (MCI). MCI is another cognitive disorder that has a wide range of types and severity of cognitive impairments, but MCI is typically regarded as a less severe cognitive disorder than dementia.^21^ Similar to BD, individuals with MCI are at risk of potentially developing dementia as their cognitive symptoms worsen over time.^22,23^ There have been some further similarities reported between cognitive impairments in BD and MCI,^24^ but there are no known, established conclusions on the similarities and differences between BD and MCI. Considering the likelihood of both MCI and BD patients developing dementia-like symptoms with the progression of each disorder, investigating the similarities and differences in severity of affected cognitive domains between the two disorders is an intriguing question that could yield important clinical information.

To our knowledge, there are no systematic reviews assessing comparisons of cognitive impairment between BD, MCI, and dementia. Investigating the similarities of affected cognitive domains and the severity of cognitive impairments in BD relative to MCI and dementia could contribute to further understanding cognitive symptoms in BD and highlight the need for early detection and prevention of these symptoms. Thus, the aim of this study was to describe the literature comparing cognitive performance in BD with MCI and/or dementia.

## Methods

The Preferred Reporting Items for Systematic Reviews and Meta-Analysis (PRISMA) guidelines were followed for the present review. The systematic review has been registered on the International Prospective Register of Systematic Reviews (PROSPERO) under registration ID: CRD42020150412.

### Search strategy

**Figure 1:**
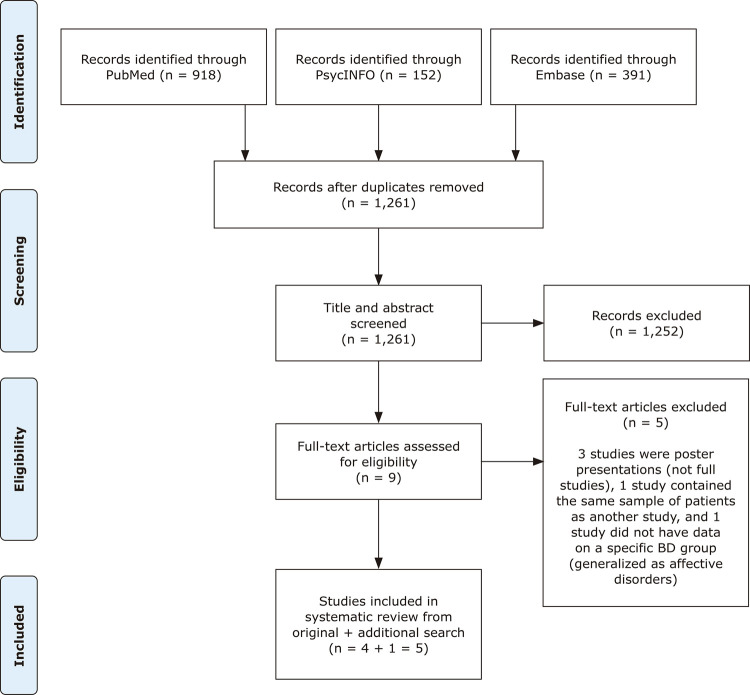
Preferred Reporting Items for Systematic Reviews and Meta-Analysis (PRISMA) flow diagram of the selection process for the inclusion of studies. BD = bipolar disorder.

A literature search was conducted on September 4, 2019, without language or year restrictions, using the following databases: PubMed, PsycINFO, and Embase. We searched for a combination of the following search terms: (Bipolar disorder OR Bipolar Disorders OR Mania OR Bipolar Affective Disorders OR Manic-Depressive Psychosis) AND (Mild cognitive impairment OR Mild cognitive impairments OR Mild Neurocognitive Disorder OR Mild Neurocognitive Disorders OR dementia) AND (cognitive complaints OR neurocognitive performance OR cognitive impairment). The searches yielded 1461 articles: (PubMed = 918, PsycINFO = 152, Embase = 391) and 1,261 articles remained after removal of duplicates (Figure 1).

We used the following inclusion criteria to determine whether an article was relevant to our study: the study should 1) present original data, 2) include patients with BD, 3) and compare them to subjects with MCI or dementia, 4) with regards to cognitive complaints/cognitive performance. The exclusion criteria were 1) reviews, 2) meta-analyses, and 3) case-reports.

The studies were selected by two blinded reviewers (MS and AM) who determined if studies met the inclusion criteria. Articles were assessed independently by both raters and disagreements were resolved by consensus in a meeting with a third researcher (TAC). First the raters screened articles based on their titles and abstracts and then they screened articles selected as relevant by reading the full text. Duplicates, review articles, and articles that did not fulfill the inclusion criteria were excluded.

### Data extraction

Two researchers (MS and AM) conducted the data extraction process. The following data were extracted: authorship, year of publication, journal, country, aim of the study, population included in the study, study design, assessments, and main results of the study. All cognitive domains were included in extraction and analysis, provided they were assessed using valid cognitive tests.

### Quality assessment

Each manuscript included in the review was independently assessed by two blinded researchers (MS and AM) using the Newcastle-Ottawa Quality Assessment Scale (NOQAS).^25^ Disagreements were resolved by consensus in a meeting with a third researcher (TAC).

## Results

The literature search yielded 1,461 articles. Once duplicates had been removed, 1,261 papers remained. We excluded 1,251 studies based on the titles and abstracts and another five articles based on full-text screening. Additionally, we hand-searched the references of the studies included from the database searches and found one additional study that fit the inclusion criteria. This resulted in a final total of five studies to be included in the systematic review. Figure 1 illustrates the paper selection and inclusion process.

### Characteristics of studies included

Table 1 shows an overview of the studies included. The publication dates of the five studies ranged from 2009 to 2017. One study was conducted in Brazil, one in the Netherlands, one in Argentina, one in Portugal, and one in Israel. Total sample sizes ranged from 29^26^ to 214^27^ and mean age ranged from 41 to 71 years.

**Table 1:** Characteristics of all studies included

Author, journal, country	Aim	Diagnostic criteria	Population^†^	Study design	Assessments	Main results
Silva,^24^ International Journal of Geriatric Psychiatry, Portugal	To characterize cognitive deficits of patients with BD in comparison to MCI	BD = DSM-IV MCI = criteria from the MCI Working Group of the European Consortium on Alzheimer’s disease	BD = 45 (64.4); age: 63.8 (8.8); years of education: 10.0 (4.4) MCI = 90 (64.4); age: 64.2 (8.4); years of education: 8.4 (4.3)	Cross-sectional	BLAD MMSE CDR BDRS GDS	MCI > BD in attention, motor initiative, verbal abstraction and calculation tasks BD > MCI in episodic memory tasks
Baez,^26^ Neuropsychologia, Argentina	To compare executive functioning, social cognition profiles, and structural neuroimaging between bvFTD and elderly patients with BD	BD = DSM-IV bvFTD = clinical meeting with experts such as cognitive neurologists, psychiatrists, and neuropsychologists	BD = 13 (76.9); age: 61.9 (9.1); years of education: 14.6 (4.5) bvFTD = 16 (56.2); age: 65.8 (7.0); years of education: 14.8 (4.3)	Cross- sectional	MMSE IFS Backward Digit Span TMT WCST RMET Letter fluency (verbal)	MMSE: BD > bvFTD (< 0.05) Executive function (IFS): BD > bvFTD (< 0.05) Verbal working memory: BD > bvFTD (< 0.05) WCST: BD > bvFTD (< 0.05) TMT: BD > bvFTD (< 0.001) RMET: BD > bvFTD (< 0.001)
Vijverberg,^27^ Journal of Clinical Psychiatry, Netherlands	To compare neuropsychological profiles in bvFTD and BD in older patients with active symptoms	BD = DSM-IV for current (hypo)manic or depressive episode bvFTD = standardized 1-day assessment (medical history, neurological, cognitive, and neuropsychological assessments) by a neurologist	BD = 41 (53.6); age: 71.7 (8.8); years of education: N/A bvFTD = 173 (40.3); age: 62.6 (8.0); years of education: N/A	Cross-sectional	MMSE Digital span test TMT A & B RAVLT Stroop Test Naming Fluency Test	Executive function: bvFTD >** BD Attention and working memory: bvFTD >** BD Verbal memory: bvFTD > **BD Verbal fluency: BD > ** bvFTD Cognitive performance: BD > ** bvFTD
						
Osher,^28^ Psychotherapy and Psychosomatics, Israel	To compare psychological functioning of euthymic BD patients vs. MCI patients	BD = DSM-IV MCI = expert clinical examination independent of neurocognitive test scores	BD = 51 (50.9); age: 41.3 (13.2); years of education: 12.8 (2.0) MCI = 162 (43.2); age: 72.8 (8.7); years of education: 13.0 (3.6)	Cross-sectional	GAB	Women with MCI > women with BD in visual-spatial processing, attention, and motor skills
Aprahamian,^29^ American Journal of Geriatric Psychiatry, Brazil	To investigate performance on cognitive screening tests in a sample of older adults with BD, as compared to non-BD subjects	BD = DSM-IV Dementia = DSM-IV CIND = multidisciplinary consensus (followed Petersen’s criteria for MCI)	BD (no CIND) = 35 (65.7); age: 68.6 (6.4); years of education: 11.4 (3.6) BD (CIND) = 25 (64); age: 66.8 (3.6); years of education: 8.8 (5.4) AD = 30 (73.3); age: 70.9 (6.2); years of education: 5.1 (3.4)	Cross-sectional	CAMCOG MMSE VFT CDT	CAMCOG: BD (both) >** AD MMSE: BD (both) >** AD VFT: a) BD >** AD; b) BD-CIND >*AD CDT: a) BD >** AD; b) BD-CIND = AD

AD = Alzheimer’s disease; BD = bipolar disorder; BDRS = Blessed Dementia Rating Scale; BLAD = Battery of Lisbon for the Assessment of Dementia; bvFTD = behavioral variant frontotemporal dementia; CAMCOG = Cambridge Cognitive Test; CDR = Clinical Dementia Rating Scale; CDT = Clock Drawing Test; CIND = cognitive impairment without dementia; GAB = Global Assessment Battery; GDS = Geriatric Depression Scale; IFS = INECO frontal screening; MCI = mild cognitive impairment; MDD = major depressive disorder; MMSE = Mini Mental State Examination; N/A = not available; RAVLT = Rey Auditory Verbal Learning Test; RMET = Reading the Mind in the Eyes Test; TMT = Trail Making Test; VFT = Verbal Fluency Test; WCST = Wisconsin Card Sorting Test.

^†^ Sample size (% females); mean age (standard deviation [SD]); mean years of education (SD); * < 0.05; ** < 0.01; > a better score on the assessments, indicating less impairment.

Two studies compared cognitive performance between BD and MCI, and three studies assessed cognitive performance between BD and dementia. All five studies had cross-sectional designs. Regarding the assessments used for inclusion criteria, all studies used DSM-IV criteria for diagnosis of BD, two studies examined MCI patients through specific clinical evaluations for MCI, one study used DSM-IV criteria for Alzheimer’s disease (AD) dementia, and two used specific clinical examinations by experts for behavioral variant frontotemporal dementia (bvFTD) (Table 1).

### Quality assessments of the included studies

Each study included in this review was examined and critically appraised using the adapted NOQAS for cross-sectional studies.^25^ Results are shown in Table 2. The maximum score on the scale for cross-sectional studies is 10 and the scores of the studies included ranged from 9 to 10, with a mean score of 9.4. All five of the studies included were rated as high quality.

**Table 2:** Quality assessment of the studies included using the Newcastle-Ottawa Quality Assessment Scale (NOQAS)

Author	Representativeness of the sample (selection bias)	Sample size (selection bias)	Non-respondents (selection bias)	Ascertainment of exposure (selection bias)	Comparability (comparability bias)	Assessment of outcome (outcome bias)	Statistical test (outcome bias)	Total score
Silva^24^	1	1	1	2	2	2	1	10/10
Baez^26^	1	1	1	2	2	2	1	10/10
Vijverberg^27^	1	-	1	2	2	2	1	9/10
Osher^28^	1	-	1	2	2	2	1	9/10
Aprahamian^29^	1	-	1	2	2	2	1	9/10

### Cognitive performance between bipolar disorder and mild cognitive impairment

Two studies assessed cognitive performance between BD and MCI^24,28^ and the main results showed that BD subjects demonstrated greater cognitive impairment than MCI subjects, especially in the following specific areas: attention,^24,28^ motor initiative,^24,28^ conceptual thinking,^24^ calculation,^24^ and visual-spatial abilities.^28^ On the other hand, MCI subjects were significantly more impaired in tests of immediate and delayed recall (logical memory)^17^ than individuals with BD.

Silva et al.^24^ conducted a cross-sectional study including 45 individuals with BD during euthymia (mean age 63.8±8.8) and 90 individuals with MCI (mean age 64.2±8.4). BD was diagnosed using DSM-IV criteria and subjects in a current acute mood episode were excluded from the study. Individuals with MCI were assessed according to the criteria defined by the MCI Working Group of the European Consortium on Alzheimer’s Disease.^30^ In this study, the Battery of Lisbon for the Assessment of Dementia (BLAD) was used to assess the participants in various cognitive domains such as: attention and executive functions; verbal, motor, and graphomotor initiatives; verbal comprehension; verbal and non-verbal abstraction; visuo-constructional abilities; calculation; immediate memory; working memory; learning and verbal memory. The findings reported indicated more severe impairments in the BD group in comparison to the MCI group in the following assessments: attention and executive functions (cancellation task) (p = 0.02), motor initiative (p < 0.01), conceptual thinking (interpretation of proverbs) (p = 0.02), and basic calculations (p = 0.01). In contrast, the MCI group exhibited more severe impairment in logical memory (immediate and delayed recall) (p < 0.01) than the BD group. These results were adjusted for the presence of depressive symptoms.

Osher et al.^28^ also conducted a cross-sectional study aimed at comparing the cognitive performance between individuals with BD during euthymia and individuals with MCI. Subjects with BD (n = 51, mean age: 41.3±13.2) had to be euthymic for at least a month prior to the study. Subjects with MCI (n = 162, mean age: 72.8±8.7) were assessed based on a clinical examination independent from the neurocognitive tests of the study. A computerized cognitive assessment battery known as the Global Assessment Battery (GAB) was used in this study. The assessment consists of 10 different tests, examining seven different cognitive domains: memory, executive function, visual-spatial processing, verbal function, attention, information processing speed, and motor skills. Considering the whole sample, BD and MCI subjects demonstrated statistically equivalent scores on the memory, executive function, verbal function, and information processing speed tests. However, differences were found between BD and MCI when stratifying the sample by sex. Women with BD (n = 26) demonstrated more severe impairments in visual-spatial processing (p = 0.0078), attention (p = 0.0066), and motor skills (p = 0.0098) than women with MCI (n = 92). There were no differences between men with BD and men with MCI. Additionally, the effect of different medication treatments on cognitive function in BD was also assessed. There were no differences in neurocognitive performance between patients with BD on mood stabilizer monotherapy, neuroleptic monotherapy, or polypharmacy.

### Cognitive performance between bipolar disorder and dementia

We included three studies assessing cognitive performance between BD and dementia.^26,27,29^ Two out of the three studies included compared BD to bvFTD,^26,27^ while one study compared euthymic BD to AD.^29^ One of the two studies comparing BD and bvFTD only included patients with BD in a current (hypo)manic or depressive episode,^27^ while the other study only included euthymic BD patients.^26^ The main finding consistent across all three studies was that overall cognitive impairment was greater in dementia than in BD, considering the total scores of the cognitive screening tests used in the studies, such as the Cambridge Cognitive Test (CAMCOG)^29^ and the Mini Mental State Examination (MMSE).^26,27,29^ Patients with both types of dementia (bvFTD and AD) indicated greater general and specific cognitive impairments than euthymic patients with BD across two studies,^26,29^ while, intriguingly, subjects with BD currently experiencing a mood episode presented greater impairments than bvFTD in several specific cognitive areas, such as attention, working memory, verbal memory, and executive function.^27^ It is important to highlight that the differences in cognitive functioning in specific areas in BD relative to dementia could be associated with the current mood state of individuals with BD.

Baez et al.^26^ conducted a study including 13 euthymic patients with BD (mean age: 61.9±9.1 years), and 16 subjects with bvFTD (mean age: 65.8 ± 7.0 years). Patients with BD were diagnosed using DSM-IV criteria, while subjects with bvFTD were evaluated through meetings with clinical experts following a standard examination battery including neurological, neuropsychiatric, and neuropsychological examinations. This cross-sectional study compared executive and social cognitive functioning as well as the structural brain differences between the two groups. General cognitive functioning was assessed using the MMSE, where bvFTD subjects reported greater overall cognitive impairments than BD subjects (p < 0.05). Specific cognitive evaluations were conducted using the INECO frontal screening (IFS) test, which consists of eight subtests of motor programming, interference, motor inhibitory control, numerical working memory (backward digit span), verbal working memory, spatial working memory, abstraction capacity, and verbal inhibitory control. In addition, participants also completed tests of verbal fluency, trail making tests (TMT) for attention and mental flexibility, the Modified Wisconsin Card Sorting Test (WCST) for abstraction capacity and executive functioning, and the Reading the Mind in the Eyes Test (RMET) for theory of mind evaluation. The findings from these evaluations suggested more severe impairments in bvFTD in comparison to BD in the following tests: IFS total score (p < 0.05), IFS – verbal working memory (p < 0.05), WCST (p < 0.05), TMT (p < 0.001), and RMET (p < 0.001). There were no significant differences between the groups in the remaining tests used in this study. In summary, findings from this study suggest greater cognitive impairments in bvFTD relative to euthymic individuals with BD across several cognitive domains, such as overall cognitive stability, executive functioning, attention, verbal working memory, and theory of mind. In contrast, they found similar performances between bvFTD and euthymic BD participants across several cognitive domains.

Vijverberg et al.^27^ performed a cross-sectional study including 41 non-euthymic participants with BD (mean age: 71.7±8.8 years) and 173 subjects with bvFTD (mean age: 62.6±8.0 years). Twenty of the 41 subjects with BD were in a manic state and 21 were in a depressive state at the period of assessment, as assessed using DSM-IV criteria. Subjects with bvFTD were diagnosed with a standardized one-day evaluation of medical history and neurological, cognitive, and neuropsychological assessments conducted by a neurologist. The study compared neuropsychological profiles between the two groups. All analyses were adjusted for age, gender, and education level. All neuropsychological data were transformed into z scores standardized to the average performance of the healthy controls in that assessment. Overall cognitive well-being was assessed using the MMSE, which indicated significantly more severe impairments in BD than in bvFTD subjects (p < 0.001). Furthermore, different cognitive domains were evaluated using specific cognitive assessments, such as attention and working memory (digit span forward, digit span backward, TMT-A), verbal memory (word learning, word retention), executive function (TMT-B, stroop interference), and verbal fluency (animal naming, letter naming). Scores in these evaluations suggested more severe impairments in BD than in bvFTD in attention and working memory (p < 0.001), executive function (p < 0.001), and verbal memory (p < 0.001), whereas bvFTD subjects demonstrated greater deficits in verbal fluency (p < 0.001) than BD subjects. The group of bvFTD subjects did significantly better than non-euthymic BD subjects in the overall and specific cognitive assessments in this study, with the exception of the verbal fluency tests (VFT).

Aprahamian et al.^29^ conducted a cross-sectional study differentiating between people with a BD diagnosis (n = 86, euthymic) and without a BD diagnosis (n = 100), as assessed by DSM-IV criteria. Furthermore, they subdivided the groups based on their cognitive abilities as evaluated by a multidisciplinary consensus which followed the same diagnostic process as Petersen’s criteria for MCI^31^ (normal cognition, cognitive impairment no dementia [CIND], or AD dementia). Patients with AD were diagnosed using DSM-IV criteria. For the purposes of our research interests (contrasting between BD and dementia), we considered two comparisons between three subgroups in this study: 1) comparison between subjects with normal cognition and BD (n = 35, mean age 68.6±6.4) and with AD but without BD (n = 30, mean age 74±8.4); and 2) a comparison between BD-CIND subjects (n = 25, mean age 66.8±3.6) and the same AD group included in the first comparison. The total sample size from this study considered in our systematic review was 90 patients. All participants were assessed with the Brazilian version of the CAMCOG. The CAMCOG measures performance in orientation, memory, attention, praxis, calculation, abstract thinking, and perception. Subjects were also evaluated according to scores on the MMSE, clock-drawing test (CDT), and VFT. For the purpose of this systematic review, we performed additional analyses to evaluate the significance of differences between the groups of interest in scores for these assessments. The results for scores for each test are as follows:

Comparison between subjects with normal cognition BD and AD:

- CAMCOG: BD (94.2±6.3) > AD (63.4±11.6) – p < 0.001
- MMSE: BD (27.8±1.7) > AD (18.9±3.9) – p < 0.001
- VFT: BD (15.9±4.8) > AD (10.8±4.7) – p < 0.001
- CDT: BD (4.2±0.6) > AD) (2.7±1.0) – p < 0.001

The BD group performed significantly better than the AD group on all the assessments in this study, indicating more severe cognitive impairment in the AD group in comparison to the BD group.

Comparison between BD-CIND and AD subjects:

- CAMCOG: BD (86.5±7.2) > AD (63.4±11.6) – p < 0.001
- MMSE: BD (25.8±2.2) > AD (18.9±3.9) – p < 0.001
- VFT: BD (14.5±3.5) > AD (10.8±4.7) – p < 0.05
- CDT: BD (3.1±0.8) > AD (2.7±1.0) – p = 0.112

The BD-CIND group performed significantly better than the AD group on all the assessments in this comparison, with the exception of the CDT, for which we did not find any differences between the groups.

In addition, patients with BD were taking lithium (40.7%), valproate (39.5%), carbamazepine (16.3%), antidepressants (47.7%), antipsychotics (27.9%), and benzodiazepines (40.7%), either isolated or in combination. No significant effect of current use of psychotropic medications was observed on cognitive test scores in normal cognition BD or BD-CIND subjects.

## Discussion

Findings from our systematic review indicate more severe cognitive deficits in euthymic BD relative to MCI, but less severe than deficits observed in dementia. Based on the two studies comparing BD and MCI, people with BD present more severe impairments in cognitive domains such as attention and executive functioning, motor skills, conceptual thinking, and visuo-spatial abilities than people with MCI. In contrast, our systematic review showed that euthymic patients with BD exhibited lower levels of overall and specific cognitive impairments than individuals with bvFTD and AD, whereas patients with BD in a current episode demonstrated more severe cognitive impairments than people with bvFTD in areas such as attention, working memory, verbal memory, and executive function. These findings suggest that cognitive deficits in euthymic BD range somewhere between those of people with MCI and those of people with dementia.

### Comparison between bipolar disorder and mild cognitive impairment

We analyzed the findings of two studies comparing cognitive deficits in euthymic BD and MCI subjects to inspect the differences and similarities between two disorders at risk of developing dementia. A common observation across both studies was that euthymic BD subjects demonstrated more severe deficits in attention and motor initiative than adults with MCI. Impairments in attention, processing speed, and executive functioning in euthymic BD have been reported in multiple studies, including a meta-analysis of those studies, suggesting these cognitive domains to be among the most commonly affected in BD.^14^ Intriguingly, the two studies analyzed in our review suggest these domains to also be more severely affected in euthymic BD in comparison to people with MCI, not just in comparison to a healthy population. In addition, psychomotor dysfunction in BD has been studied and observed in previous studies as well,^32,33^ and it is a cognitive domain included in many diagnostic assessments. Findings from our systematic review suggest that motor initiative, in particular, may be impaired to a greater extent in euthymic BD relative to MCI. In addition to familiar findings about motor deficits in comparison to healthy populations, this may yield a better understanding of the particular subpar motor movements affecting people with BD, and the extent to which they are affected relative to a similar cognitive disorder such as MCI.

Furthermore, one of the two studies reported significant deficits in conceptual thinking (interpretation of proverbs) and basic calculations in BD in comparison to MCI.^24^ The second study, conducted by Osher et al.,^28^ suggested more severe deficits in visuo-spatial processing in euthymic BD relative to MCI. These are very intriguing findings, considering these domains may not be closely investigated in common diagnostic assessments. In contrast, the study conducted by Silva et al.^24^ suggested more severe deficits in logical memory (immediate and delayed recall) in MCI in comparison to BD, which is one of the hallmark symptoms of dementia. Regarding other cognitive domains examined in these studies, there were no differences between BD and MCI in language or verbal functioning, indicating similar abilities between these two diagnostic groups.

### Comparison between bipolar disorder and dementia

As expected, we also found that older adults with dementia demonstrated significantly greater cognitive deficits than in BD subjects in euthymia in general cognitive assessments, such as the CAMCOG and MMSE, as well as in specific assessments of verbal fluency, executive function, attention, working memory, and theory of mind.^26,29^ Findings from these examinations imply that major cognitive domains are not affected in euthymic BD to the extent seen in different types of dementia (bvFTD and AD). However, an intriguing observation from the Aprahamian et al.^29^ study is the similarity in scores on the CDT between the cognitively impaired BD group and the AD subjects. The CDT is a widely used screening tool for dementia and for detection of cognitive decline.^34,35^ The similarity of scores in this comparison could hint at progressive cognitive decline in BD towards the levels of impairment seen in AD.

In contrast, in the study by Vijverberg et al.,^27^ subjects with BD who were currently experiencing either a manic or depressive episode demonstrated greater impairments in attention, memory, verbal learning, and executive function, compared to the group of people with bvFTD.^27^ It is interesting to note the difference in cognitive performances in BD during a mood episode and euthymia, relative to cognition in bvFTD. Frontotemporal dementia is far less common than AD, with more than half of affected individuals experiencing behavioral changes such as interpersonal skills, executive dysfunction, and emotional dysregulation.^36^ Findings from this systematic review suggest that the severity of cognitive impairments in bvFTD ranges between those seen in euthymic BD and in BD during a mood episode. Further investigations should be conducted analyzing the similarities and differences in this fairly new cognitive comparison between BD and bvFTD.

The differences in cognitive performance in BD during euthymia and an acute mood episode have previously been observed in several studies.^12,37^ People with BD who are currently in a manic or depressive episode display higher cognitive impairments than euthymic people with BD, particularly in cognitive areas such as verbal learning, memory, verbal fluency, and executive function.^12,37^ Furthermore, current findings from this review scale the differences in cognitive deficits across different mood stages in BD relative to deficits in MCI and dementia. Considering the importance of assessing cognitive abilities in BD during euthymia, analyses of the findings from the five studies in this review indicate that cognitive impairments seen in BD are more severe than deficits in MCI, but not affected to the extent of deficits seen in bvFTD and AD dementia. In contrast, further investigations should be conducted focusing on the comparison between BD during a mood episode and dementia, with findings from the Vijverberg et al.^27^ study reinforcing previous suggestions of significant variability in cognitive abilities across different mood stages in BD.

### Limitations and strengths

The findings reported in this review should be interpreted considering some limitations. Our major limitation was the small number of studies included in the review. A limited number of studies satisfied the inclusion criteria because of their specificity. Also, the studies included in this systematic review were conducted with small samples. In addition, the studies included were heterogeneous in terms of sample selection. Lastly, some of the studies included did not consider the potential effects of participants’ medications on their cognitive abilities, although it is known that medications for patients with BD could have undesired side effects in several cognitive domains.^38^ Despite these limitations, this is the first systematic review comparing cognitive performance in BD, MCI, and dementia and the studies included were all rated as high quality as per the NOQAS quality assessment tool.

## Conclusion

To our knowledge, this is the first systematic review comparing cognitive abilities in BD in comparison to MCI and dementia. Cognitive testing of people with BD in comparison to people with MCI could imply that cognitive dysfunction in BD exceeds the cognitive deficits seen in MCI, even during the remission phase in BD. The clinical outcomes of the progression of BD are evident through the cognitive decline throughout the course of the disorder.^39^ However, the studies included in this systematic review did not separate subjects with BD according to their clinical stages. Patients in later stages of BD typically demonstrate greater cognitive deficits than early-stage patients, due to progression of the disorder.^40,41^ Presumably, cognition in late-stage BD might be more similar to cognition in MCI or dementia than cognition in early-stage BD.

In conclusion, future studies should analyze the cognition of people with BD according to their clinical stage (early vs. late-stage) in comparison to other cognitive disorders, such as MCI and dementia. Differentiation by clinical stages of BD would highlight the progression of clinical outcomes in this disorder, indicating the progression of cognitive symptoms towards those of other cognitive disorders. Future investigations should also focus on proper categorization of subjects based on their current mood state and subtype of diagnosis, as there may be differences in cognitive functionality across different mood states and subtypes. Nevertheless, deficits in cognitive domains such as attention, motor initiative, visual perception and executive function are evident in people with BD. The clinical outcomes of the disorder could result in detrimental effects on the cognitive functioning of people with BD, resulting in cognitive deficits similar to those of MCI and dementia.

## References

[1] Grande I, Berk M, Birmaher B, Vieta E (2016). Bipolar disorder. Lancet.

[2] Merikangas KR, Jin R, He JP, Kessler RC, Lee S, Sampson NA (2011). Prevalence and correlates of bipolar spectrum disorder in the world mental health survey initiative. Arch Gen Psychiatry.

[3] Jansen K, Magalhães PVS, Pinheiro RT, Kapczinski F, Silva RA (2012). Early functional impairment in bipolar youth: a nested population-based case-control study. J Affect Disord.

[4] Rosa AR, Bonnín CM, Vázquez GH, Reinares M, Solé B, Tabarés-Seisdedos R (2010). Functional impairment in bipolar II disorder: is it as disabling as bipolar I?. J Affect Disord.

[5] Jansen K, Campos Mondin T, Azevedo Cardoso T de, Costa Ores L da, Mattos Souza LD, Tavares Pinheiro R (2013). Quality of life and mood disorder episodes: community sample. J Affect Disord.

[6] Solé B, Jiménez E, Torrent C, Reinares M, Bonnin CDM, Torres I (2017). Cognitive impairment in bipolar disorder: treatment and prevention strategies. Int J Neuropsychopharmacol.

[7] Cardoso T, Bauer IE, Meyer TD, Kapczinski F, Soares JC (2015). Neuroprogression and cognitive functioning in bipolar disorder: a systematic review. Curr Psychiatry Rep.

[8] Sparding T, Silander K, Pålsson E, Östlind J, Ekman CJ, Sellgren CM (2017). Classification of cognitive performance in bipolar disorder. Cogn Neuropsychiatry.

[9] Goldberg JF, Chengappa KNR (2009). Identifying and treating cognitive impairment in bipolar disorder. Bipolar Disord.

[10] Zarate CA, Tohen M, Land M, Cavanagh S (2000). Functional impairment and cognition in bipolar disorder. Psychiatr Q.

[11] Douglas KM, Gallagher P, Robinson LJ, Carter JD, McIntosh VV, Frampton CM (2018). Prevalence of cognitive impairment in major depression and bipolar disorder. Bipolar Disord.

[12] Martínez-Arán A, Vieta E, Reinares M, Colom F, Torrent C, Sánchez-Moreno J (2004). Cognitive function across manic or hypomanic, depressed, and euthymic states in bipolar disorder. Am J Psychiatry.

[13] Volkert J, Schiele MA, Kazmaier J, Glaser F, Zierhut KC, Kopf J (2016). Cognitive deficits in bipolar disorder: from acute episode to remission. Eur Arch Psychiatry Clin Neurosci.

[14] Torres IJ, Boudreau VG, Yatham LN (2007). Neuropsychological functioning in euthymic bipolar disorder: a meta-analysis. Acta Psychiatr Scand Suppl.

[15] MacQueen GM, Young LT, Robb JC, Marriott M, Cooke RG, Joffe RT (2000). Effect of number of episodes on wellbeing and functioning of patients with bipolar disorder. Acta Psychiatr Scand.

[16] Cao B, Passos IC, Mwangi B, Bauer IE, Zunta-Soares GB, Kapczinski F (2016). Hippocampal volume and verbal memory performance in late-stage bipolar disorder. J Psychiatr Res.

[17] Diniz BS, Teixeira AL, Cao F, Gildengers A, Soares JC, Butters MA (2017). History of bipolar disorder and the risk of dementia: a systematic review and meta-analysis. Am J Geriatr Psychiatry.

[18] Kessing LV, Andersen PK (2004). Does the risk of developing dementia increase with the number of episodes in patients with depressive disorder and in patients with bipolar disorder?. J Neurol Neurosurg Psychiatry.

[19] Blazer D (2017). Bipolar disorder and dementia: weighing the evidence. Am J Geriatr Psychiatry.

[20] Velosa J, Delgado A, Finger E, Berk M, Kapczinski F, Azevedo Cardoso T (2020). Risk of dementia in bipolar disorder and the interplay of lithium: a systematic review and meta-analyses. Acta Psychiatr Scand.

[21] Sanford AM (2017). Mild Cognitive impairment. Clin Geriatr Med.

[22] Ravaglia G, Forti P, Maioli F, Martelli M, Servadei L, Brunetti N (2006). Conversion of mild cognitive impairment to dementia: predictive role of mild cognitive impairment subtypes and vascular risk factors. Dement Geriatr Cogn Disord.

[23] Roberts RO, Knopman DS, Mielke MM, Cha RH, Pankratz VS, Christianson TJH (2014). Higher risk of progression to dementia in mild cognitive impairment cases who revert to normal. Neurology.

[24] Silva D, Santana I, do Couto FS, Maroco J, Guerreiro M, Mendonça A (2009). Cognitive deficits in middle-aged and older adults with bipolar disorder and cognitive complaints: comparison with mild cognitive impairment. Int J Geriatr Psychiatry.

[25] Stang A (2010). Critical evaluation of the Newcastle-Ottawa scale for the assessment of the quality of nonrandomized studies in meta-analyses. Eur J Epidemiol.

[26] Baez S, Pinasco C, Roca M, Ferrari J, Couto B, García-Cordero I (2019). Brain structural correlates of executive and social cognition profiles in behavioral variant frontotemporal dementia and elderly bipolar disorder. Neuropsychologia.

[27] Vijverberg EGB, Schouws S, Meesters PD, Verwijk E, Comijs H, Koene T (2017). Cognitive deficits in patients with neuropsychiatric symptoms: a comparative study between behavioral variant frontotemporal dementia and primary psychiatric disorders. J Clin Psychiatry.

[28] Osher Y, Dobron A, Belmaker RH, Bersudsky Y, Dwolatzky T (2011). Computerized testing of neurocognitive function in euthymic bipolar patients compared to those with mild cognitive impairment and cognitively healthy controls. Psychother Psychosom.

[29] Portet F, Ousset PJ, Visser PJ, Frisoni GB, Nobili F, Scheltens P (2006). Mild cognitive impairment (MCI) in medical practice: a critical review of the concept and new diagnostic procedure. Report of the MCI Working Group of the European Consortium on Alzheimer’s Disease. J Neurol Neurosurg Psychiatry.

[30] Aprahamian I, Ladeira RB, Diniz BS, Forlenza OV, Nunes PV (2014). Cognitive impairment in euthymic older adults with bipolar disorder: a controlled study using cognitive screening tests. Am J Geriatr Psychiatry.

[31] Petersen RC, Doody R, Kurz A, Mohs RC, Morris JC, Rabins PV (2001). Current concepts in mild cognitive impairment. Arch Neurol.

[32] Morsel AM, Temmerman A, Sabbe B, Hulstijn W, Morrens M (2015). Unraveling psychomotor slowing in bipolar disorder. Neuropsychobiology.

[33] King JB, Anderson JS, Yurgelun-Todd DA, Subramaniam P, Ehrler MR, Lopez-Larson MP (2018). Decreased anterior cingulate activation in a motor task in youths with bipolar disorder. J Child Psychol Psychiatry.

[34] Nair AK, Gavett BE, Damman M, Dekker W, Green RC, Mandel A (2010). Clock drawing test ratings by dementia specialists: interrater reliability and diagnostic accuracy. J Neuropsychiatry Clin Neurosci.

[35] Shulman KI (2000). Clock-drawing: is it the ideal cognitive screening test?. Int J Geriatr Psychiatry.

[36] Warren JD, Rohrer JD, Rossor MN (2013). Frontotemporal dementia. BMJ.

[37] Dixon T, Kravariti E, Frith C, Murray R, McGuire P (2003). Effect of symptoms on executive function in bipolar illness. Schizophr Res.

[38] Dias VV, Balanzá-Martinez V, Soeiro-de-Souza MG, Moreno RA, Figueira ML, Machado-Vieira R (2012). Pharmacological approaches in bipolar disorders and the impact on cognition: a critical overview. Acta Psychiatr Scand.

[39] Schouws SN, Comijs HC, Dols A, Beekman ATF, Stek ML (2016). Five-year follow-up of cognitive impairment in older adults with bipolar disorder. Bipolar Disord.

[40] Van Rheenen TE, Lewandowski KE, Bauer IE, Kapczinski F, Miskowiak K, Burdick KE (2020). Current understandings of the trajectory and emerging correlates of cognitive impairment in bipolar disorder: an overview of evidence. Bipolar Disord.

[41] Kessing LV, Andersen PK (2017). Evidence for clinical progression of unipolar and bipolar disorders. Acta Psychiatr Scand.

